# Natriuretic peptides regulate sympathetic nervous activity independent of mineralocorticoid receptor

**DOI:** 10.1186/2050-6511-16-S1-A71

**Published:** 2015-09-02

**Authors:** Hitoshi Nakagawa, Yasuki Nakada, Yoshihiko Saito

**Affiliations:** 1 First Department of Internal Medicine, Nara Medical University, Kashihara, Japan

## Background

Natriuretic peptides (ANP/BNP) increase cGMP and exert cardiovascular protective effects via guanylyl cyclase A (GC-A) receptor, which is distributed in many organs such as the heart, the vasculature and the brain [1]. Sympathetic nervous system (SNS) as well as renin-angiotensin-aldosterone-system contributes to cardiovascular disease. However, the endogenous effect of GC-A signaling on SNS is not investigated. Recent study shows that activated mineralocorticoid receptor (MR) in the hypothalamus induces systemic SNS activation [2], whereas ANP infusion in human inhibited SNS activity in the heart [3]. Notably, it is reported that ANP counteracts the deleterious effects of MR in the heart [4]. Therefore, we hypothesized that ANP suppresses MR activation in the brain and leads to the inhibition of SNS activity.

## Purpose

To investigate whether ANP/GC-A signaling inhibits SNS activity through the suppression of the brain MR, we examined urinary catecholamine secretion in global GC-A receptor KO mice and the effect of intracerebroventricular (ICV) infusion of MR blocker.

## Methods and results

We measured blood pressure (BP) and urinary *norepinephrine (U-NE)* secretion in wild type and global GC-A KO mice. Both BP and U-NE is significantly higher in GC-A KO than in wild type mice, indicating SNS is activated in GC-A KO mice. To study whether SNS activation is caused by the brain MR, we infused Eplerenone (MR blocker) into the ICV with osmotic mini pump for 2 weeks. Contrary to our hypothesis, both BP and U-NE did not change after 2 weeks ICV infusion, suggesting that activated SNS in GC-A KO is independent of MR. Furthermore, high sodium diet (NaCl 6%) for 2 weeks did not increase BP and U-NE in GC-A KO mice. MR protein expression in the hypothalamus was almost similar between GC-A KO and Wild type mice. These data suggest that SNS activity in GC-A KO mice is independent of MR and insensitive to sodium load. Unexpectedly, the most of GC-A KO mice died after ICV infusion of Losartan (AT1 receptor blocker), whereas wild type mice survived.

## Conclusion

Natriuretic peptides/GC-A signaling regulates SNS activity independent of both brain MR and sodium load. Brain AT1 receptor might be important in the regulation of cardiovascular system in global GC-A KO mice.
